# Biological Significance of HCV in Various Kinds of Lymphoid Cells

**DOI:** 10.1155/2012/647581

**Published:** 2012-02-22

**Authors:** Yasuteru Kondo, Yoshiyuki Ueno, Tooru Shimosegawa

**Affiliations:** Division of Gastroenterology, Tohoku University Hospital, 1-1 Seiryo-Machi, Aoba-ku, Sendai City, Miyagi 980-8574, Japan

## Abstract

It has been reported that HCV can infect not only hepatocytes but also various kinds of lymphoid cells. Although many reports have described the biological significance of lymphotropic HCV, the issue remains controversial since the target lymphoid cells might have various kinds of functions in the immune system. One of the important roles of lymphoid cells in HCV replication is being a reservoir of HCV. Several groups described the detection of HCV-RNA in lymphoid cells after HCV eradication in plasma. Another important role of lymphotropic HCV is that it acts as a carcinogenic agent and induces immune dysfunction. In this paper, we summarize the reports regarding the biological significance of lymphotropic HCV in representative lymphoid cells.

## 1. Introduction

Hepatitis C virus (HCV) infects about 170 million people worldwide causing chronic hepatitis, liver cirrhosis, hepatocellular carcinoma, B-cell lymphoma, cryoglobulin-related disease, and various kinds of autoimmune diseases [1–3]. HCV is basically a hepatotropic virus that causes liver disease. However, HCV replication is detected not only in hepatocytes but also in B cells, T cells, monocytes, dendritic cells (DCs), and peripheral blood mononuclear cells [4–13]. The existence of HCV strains with preferential lymphocyte tropism suggests the potential role of B cells and T cells as an HCV reservoir [14–17]. Moreover, the existence of HCV in lymphoid cells could contribute to various B-cell proliferative disorders including B-cell lymphoma and mixed cryoglobulinemia and dysregulation of the cellular and humoral immune responses that have major roles in the immunopathogenesis of HCV persistent infection [18–28]. HCV infects hepatocytes, lymphoid cells, and probably other cells through CD81 and several receptor candidates. The expression of CD81 could be detected in various types of cells, including hepatocytes, B cells, T cells, and monocytes, indicating that these types of cells are potential targets of HCV infection [13]. Previously, Sung et al. reported that HCV persistently produced from a particular B-cell line (SB), which was established from an HCV-positive B-cell lymphoma, can infect and replicate in established B-cell lines (Raji, Daudi) and primary B lymphocytes [23]. Moreover, we reported that two T-cell lines (Molt-4 and Jurkat) and primary naïve T lymphocytes were infected with SB culture supernatant [13, 27, 28]. Machida et al. reported that HCV replication in B lymphocytes could contribute to the carcinogenesis of B cells based on this useful in vitro system [29, 30]. Not only Machida's group but also many groups reported the evidence of HCV replication in lymphoid cells [18, 31–38]. Various studies about the biological significance of HCV infection in lymphoid cells have been reported, although there were some negative reports regarding HCV replication in lymphoid cells [39–41]. Therefore, understanding the various effects of lymphotropic HCV is needed to treat the extrahepatic manifestations of HCV infection. In this paper, we summarize the various studies showing evidence of HCV replication and discuss the biological significance of HCV replication in lymphoid cells according to the various subsets of lymphoid cells.

## 2. Biological Significance of HCV Infection in B Lymphocytes

Many reports indicating the existence of HCV in B lymphocytes and B-cell lymphoma have been published, and many of these focused on the relevance of HCV infection to B-cell proliferative diseases including B-cell lymphoma and cryoglobulinemia [18, 23, 42, 43]. Evidence of HCV infection in B lymphocytes could be detected using PCR-based methods. The detection of negative-strand HCV-RNA was widely used to prove the replication of HCV in the cells. However, the amount of negative-strand HCV-RNA is usually quite low in comparison to positive-strand HCV-RNA [28]. Therefore, many groups including us used nested PCR with rtTh polymerase that could reduce the risk of false-positive detection [13, 27, 28, 31, 44]. In addition to nested PCR for the detection of negative-strand HCV-RNA, immunostaining of HCV individual proteins was used to detect lymphocytes with HCV replication [28, 45]. False positive and negative amplification of HCV RNA frequently occurred due to the very little amount of HCV-RNA as compared with the large excess of total cellular RNA, resulting in underestimation of viral RNA copies. Underestimation of RNA copies and wide standard deviation were observed in intracellular HCV RNA quantification by conventional real-time PCR, particularly with very low amount of HCV-RNA (10^2^ to 10^4^ copies/ug) [28]. On the other hand, the HCV-NS3 protein could be detected over 50% of B cell line with lymphotropic HCV infection [45]. Discrepancies of these data might be due to the detection methods. Therefore, we need to consider the character of detection methods and detection limit carefully.

Sung et al. established B-cell lymphoma cell lines persistently infected with hepatitis C virus in vivo and in vitro [23]. The cell lines continuously produce infectious HCV virions in culture. The virus particles produced from the culture had a buoyant density of 1.13 to 1.15 g/mL in sucrose and could infect primary human peripheral blood mononuclear cells (PBMCs) and an established B-cell line in vitro [23]. This lymphotropic HCV strain was useful to investigate the biological significance of HCV replication in lymphoid cells.

**Figure 1:**
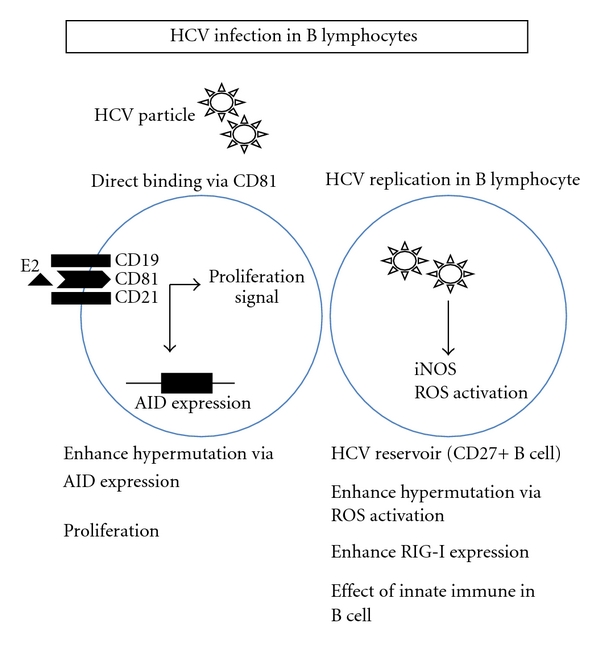
A schema of the biological significance of HCV replication in B cells is shown. The representative effects of lymphotropic HCV on B cells are shown in this figure.

It has been reported that the replication of HCV in B lymphocytes could induce error-prone DNA polymerase zeta, polymerase iota, and activation-induced cytidine deaminase (AID), which contribute to enhancing the mutation frequency [30]. Moreover, the cellular DNA damage and mutation were mediated by nitric oxide (NO) and reactive oxygen species (ROS) [20, 29]. However, not only HCV replication in B lymphocytes but also the direct HCV-E2 CD81 interaction could induce hypermutation of the immunoglobulin gene in B cells. E2-CD81 interaction on B cells triggered the enhanced expression of AID [46]. Recently, another group reported that persistent expression of the full genome of HCV in B cells induces the spontaneous development of B-cell lymphoma in vivo [47]. They established HCV transgenic mice that express the full HCV genome in B cells and observed a 25% incidence of diffuse, large B-cell non-Hodgkin lymphomas. Moreover, it has been reported that HCV replication could enhance the RIG-I expression mediated by interferon regulatory factor-2 (IRF-2) in human peripheral blood B lymphocytes. These reports explained the mechanism of carcinogenesis in lymphocytes. However, the relation between lymphotropic HCV and mixed cryoglobulinemia, one of the lymphoproliferative diseases, has been analyzed by many groups [3, 48–50]. Sansonno et al. reported that HCV replication could be detected especially in chronic HCV with mixed cryoglobulinemia [50]. The mechanisms of the induction of mixed cryoglobulinemia by lymphotropic HCV replication have not been clarified yet except for the induction of hypermutation of Ig-related genes and the stimulation of lymphocyte [45] (Figure 1).

It has been reported that the B lymphocyte reservoir of HCV is one of the important issues regarding the biological significance of lymphotropic HCV [17]. Recently, it was found that CD27^+^ memory B cells were more resistant to apoptosis than CD27^−^ B cells. CD27^+^ memory B cells might be an HCV reservoir for persistent infection in chronic hepatitis C patients [51]. Moreover, a possible mechanism by which the innate immune system is blocked in B cells, which is necessary for HCV to infect B cells persistently, was reported [14] (Figure 1). TANK-binding kinase-1 (TBK1) and I*κ*B kinase *ε* (IKK*ε*) are essential for IRF-3 phosphorylation, and both kinases were markedly enhanced in B cells with hepatitis C infection. However, the reduced expression of heat shock protein of 90 kDa, a TBK1 stabilizer, and the enhanced expression of SIKE, an IKK*ε* suppressor, were observed in B cells, and these might suppress the kinase activity of TBK1/IKK*ε* for IRF-3 phosphorylation in CHC patients [14].

## 3. Biological Significance of HCV Infection in T Lymphocytes

**Figure 2:**
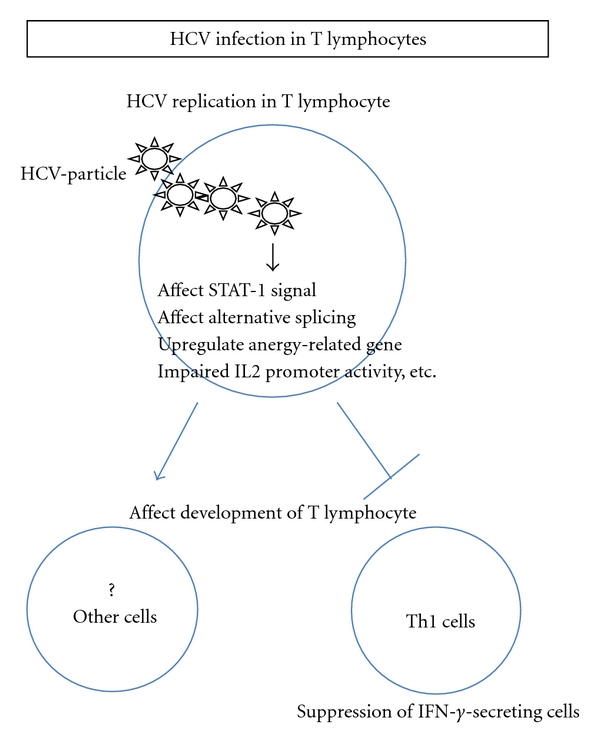
The schema of the biological significance of HCV replication in T cells is shown. The representative effects of lymphotropic HCV on T cells are shown in this figure.

Various groups reported that HCV could be detected not only in B lymphocytes but also in T lymphocytes, which could contribute to the humoral and cellular immune responses [8, 31]. Many research groups established HCV replication systems using T-cell lymphoma cell lines more than a decade ago [4, 5, 10, 11, 32]. One group reported that human T-lymphotropic virus type I infected cell line MT-2 was susceptible to HCV infection [4, 5]. Not only HTLV-I but also Epstein-Barr (EB) virus enhanced the ability of HCV to replicate in T-cell lines [35]. These reports suggested that the existence of other viruses might change the sensitivity of HCV infection in T lymphocytes. It is important to analyze the possibility that the susceptibility of HCV can be changed by other viruses, since HCV/HIV coinfection is an important issue [52, 53]. However, the biological significance of HCV replication in T lymphocytes has not been clarified yet. We previously reported that lymphotropic HCV strain (SB-HCV) could be replicated in two T-cell lines (Molt-4 and Jurkat) and primary T lymphocytes, especially in naïve CD4^+^ T lymphocytes with proliferative stimulation [13, 27, 28]. The negative strand HCV-RNA that is the evidence of viral replication could be detected temporarily after inoculation. The amount of HCV-RNA detected in T lymphocytes was lower than in B lymphocytes in this infection system. The amounts of negative strand RNA and positive-strand RNA in B cell lines were at least 4 times higher than those of T-cell lines [28]. It has been reported that depletion of CD8^+^ cells could increase the positive rate of HCV-RNA in PBMC. CD8^+^ T cells have a strong ability to produce the IFN-g that could suppress the HCV replication [54]. In our study, the negative-strand HCV-RNA could not be detected in CD8^+^ T cells. We have reported that HCV replication could affect IFN-g/STAT-1/T-bet signaling by reducing the amount of phospho-STAT-1 in Molt-4 and human primary naïve T lymphocytes [13, 28]. Moreover, HCV-replication could inhibit proliferation and enhance Fas-mediated apoptosis by downregulating the expression of CD44 splicing variant 6 [27] (Figure 2). In addition to lymphotropic SB-HCV strain, it has been reported that the wild-type HCV could infect human T lymphocytes with T-cell-stimulating mitogens [34]. Another group reported that HCV core protein upregulated anergy-related genes using a Jurkat T cell line stably expressing HCV core protein [26]. This cell line showed increased activation of NFAT transcription factor and impaired IL2 promoter activity [24] (Figure 2). The expression of HCV core in T lymphocytes might contribute to the establishment of persistent infections by inducing Ca^2+^ oscillations that regulate both the efficacy and information content of Ca^2+^ signals and are ultimately responsible for the induction of gene expression and functional differentiation [25]. Coinfection with HCV and HIV is associated with increased HCV replication and a more rapid progression to severe liver disease, including the development of cirrhosis and hepatocellular carcinoma. One group reported that HCV, HIV-1, and human herpes virus 6 were coinfected in single T cells. Coinfection of a T cell by all three viruses was confirmed by transmission electron microscopy [55].

## 4. Biological Significance of HCV Infection in Monocyte and DCs

**Figure 3:**
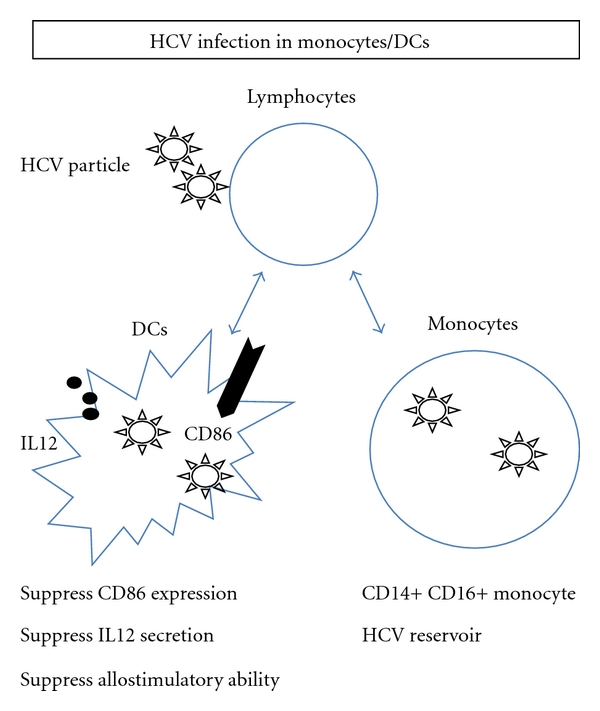
The schema of the biological significance of HCV replication in DCs and monocytes is shown. The representative effects of lymphotropic HCV on DCs and monocytes are shown in this figure.

 It has been reported that HCV could infect not only B and T lymphocytes, but also monocytes and DCs [56]. Recently, a group reported that HCV could infect CD14^+^CD16^+^ cells but not CD14^+^CD16^−^ cells. They found that one of the important HCV receptors, CD81, is highly expressed on CD14^+^CD16^+^ cells but not on CD14^+^CD16^−^ cells [57] (Figure 3). A group reported that negative strand HCV-RNA was most commonly present in monocyte/macrophages followed by T cells and B cells in 10 HCV/HIV coinfected patients [58]. Although the samples size of this study was not so large, we need to consider the significance of HCV replication in monocytes, especially in HCV/HIV co-infected patients. The interaction between monocytes and B and T lymphocytes might have an important role in the immuno-pathogenesis of HCV infection. In addition to the effects on cell function, the existence of HCV in monocytes might serve as an HCV reservoir as seen in B and T lymphocytes [15].

 Dendritic cells (DCs), which play a central role in coordinating the immune response in HCV infection, have been studied by many research groups. DCs are the most potent activators of CD4 T cells for supporting Th1 differentiation, which is important for the cellular immune response. It has been reported that DCs from chronic hepatitis C patients expressed lower amounts of CD86 and IL12 than those from healthy subjects [59]. Moreover, other groups have reported that the existence of HCV in DCs might suppress the allostimulatory function, although the detailed mechanism of suppression was not clear [60–62] (Figure 3). In vitro infection of human monocyte-derived DCs was carried out, and strand-specific rTth reverse transcription polymerase chain reaction was used to prove the HCV replication in DCs [63]. Replicative-strand RNA could be detected in 3 of 24 peripheral DCs purifications. Moreover, the analysis of the HCV quasispecies distribution in the peripheral DC population of 1 patient showed the presence of a dominant variant different from that found in plasma with respect to the primary amino acid sequence and physiological profile of the hypervariable region 1 of glycoprotein E2 [64]. However, the relationship between the dysfunction of DCs and HCV replication has been still controversial [65].

## 5. HCV Infection in Nonparenchymal Liver Cells and Other Lymphoid Cells

 Nonparenchymal liver cells include lymphocytes, kupffer, polymorphonuclear, pit, endothelial, stellate and fibroblast-like cells. One of the curious subsets of nonparenchymal liver cells is hepatic stellate cells (HSCs) that play an important role in the control of extracellular matrix synthesis and degradation in fibrotic livers. One report described that HCV-core antigen could colocalize with large lipid droplets present in HSC and with collagen fibers in the extracellular matrix. These data indicated that HCV-core antigen in the stellate cells might modulate the immune function and fibrosis [66]. However, the amount of HCV-RNA and the level of HCV replication could not be mentioned in this report. More recently, it has been reported that HCV-RNA replication could affect the gene expression of extracellular matrix-related molecules in HSC [67]. In that report, they used subgenomic HCV replicon. The average amount of HCV RNA was 3.9 copies × 10^5^/ug total RNA.

 Pluripotent hematopoietic CD34^+^ cells were another curious target of HCV replication since these cells have the ability to develop various kinds of cells. It has been reported that positive- and negative-strand HCV-RNA and HCV individual proteins could be detected in CD34^+^ hematopoietic progenitor cells [68]. The amount of negative-strand HCV-RNA was 1 × 10^3^ HCV-RNA Eq. Moreover, the existence of HCV not only in peripheral blood mononuclear cells but also in bone marrow mononuclear cells has been reported [69]. These observations are important since transplantation of allogenic CD34^+^-selected peripheral stem cells can result in the transmission of hepatitis C virus from an infected donor [70].

## 6. Concluding Remarks

Although various reports described the biological significance of HCV replication in lymphoid cells, these reports were not conclusive due to the lack of efficient in vitro culture systems. However, we should not underestimate the effect of HCV replication in lymphoid cells. In this paper, we focused on the effect of HCV replication in lymphoid cells. In addition to the role as an HCV reservoir, various reports described that HCV replication in lymphoid cells could induce dysregulation of the immune system. We need to focus not only on suppression of the immune system but also on stimulation of the immune system, since the prevalence of autoimmune diseases is much higher than in healthy subjects. A study regarding the relationship between lymphotropic HCV and autoimmune diseases is ongoing in our laboratory. As for the understanding of autoimmune diseases, HCV persistent infection might be one of the representative models of viral-induced autoimmune diseases. Recently, the technologies of deep sequencing, immunoassays with increased numbers of multicolor flow cytometry analyses, and chimera mice with human lymphocytes have been developed. These technologies, together with previous data, might be able to clarify the biological significance of lymphotropic HCV.
